# An immunocompetent lady with invasive aspergillosis presenting as disseminated lesions: a case report

**DOI:** 10.1186/s13256-024-04579-z

**Published:** 2024-08-06

**Authors:** Sher M. Sethi, Ainan Arshad

**Affiliations:** Internal Medicine, Aga Khan University Hospital, Stadium Road, Gulshan-e-Iqbal, Karachi, Pakistan

**Keywords:** Aspergillosis, Inflammation, Lesions, Lungs, Medical risk factors, Voriconazole

## Abstract

**Background:**

Invasive Aspergillosis is a fungal infection caused by Aspergillus species, typically posing life-threatening risks to immunocompromised individuals. While occurrences in immunocompetent hosts are rare, a recent case report documented fulminant pulmonary aspergillosis in an immunocompetent patient during autopsy. Here, we present a case of invasive aspergillosis in an immunocompetent woman, manifesting with disseminated lesions.

**Case presentation:**

A 29-year-old Asian woman presented to our hospital in March 2022, reporting chest pain and shortness of breath persisting for two months. Upon examination, she appeared thin and unwell, with no notable abnormalities otherwise. Radiographic imaging revealed an ill-defined lesion in her left lung. Subsequent bronchoscopy and lavage were performed, followed by initiation of empirical antibiotic therapy. Lavage results were negative for gram staining, culture, and ZN staining for AFB, but revealed numerous septate hyphae on fungal smear. Histopathological examination indicated chronic granulomatous inflammation with septal fungal hyphae, indicative of aspergillosis. Subsequent culture confirmed Aspergillus species, prompting initiation of voriconazole therapy. Remarkably, the patient exhibited significant improvement, with weight gain and restored appetite observed within a short period. Within 2 months of treatment, her symptoms resolved, and she resumed near-normal daily activities.

**Conclusion:**

This case highlights the diagnosis of aspergillosis in an immunocompetent individual presenting with disseminated nodular lesions across the lungs, mediastinum, and abdomen. Clinicians should maintain a high index of suspicion for aspergillosis in cases of non-resolving pneumonia and disseminated nodular lesions, even in patients lacking traditional predisposing factors.

## Background

Invasive Aspergillosis is a fungal infection caused by Aspergillus species. It is associated with life-threatening infections, especially in immunocompromised hosts [1]. Immunocompromised hosts are those with severe neutropenia, transplant patients on immune-suppressants and critically ill patients with prolonged use of steroids [2]. In immunocompromised individuals, invasive pulmonary aspergillosis is common. However, it rarely occurs in immunocompetent individuals [3].

There are various recent case reports of invasive aspergillosis in different parts of the body in immunocompetent hosts. A patient who was immunocompetent was found to have fulminant pulmonary aspergillosis on an autopsy report in 2018. However, there were no risk factors that could promote or enhance fungal infection in that individual [3]. Kartik *et al*. reported invasive mediastinal aspergillosis in an immunocompetent male [4]. Another case of invasive colonic aspergillosis in normal individual was reported [5]. In a study involving immunocompetent individuals with pneumonia, the prevalence of invasive pulmonary aspergillosis was found to be 3.0% [6].

We present a highly unusual case of multiple pulmonary nodular lesions extending to the mediastinum and abdomen. This emphasize and taught us that infections which are not improving with antibiotics should be evaluated further. Fungal infections should be in our differentials when dealing with such kind of patients.

## Case presentation

A 29 year old Asian woman, presented with chest pain and worsening difficulty in breathing for the past two months presented herself to our hospital in March 2022. She described feeling dyspneic during routine physical activity and becoming increasingly severe over time. She denied wheezing, coughing, orthopnea, paroxysmal nocturnal dyspnea, and palpitations. There was a low grade fever, no chills or rigors, and it was undocumented. There were chest pains at the lower ribs on both sides, which were aggravated by deep inspiration. Past medical history was insignificant. Family history was negative for any chronic disease. She is married with 2 kids. She denies history of tobacco or alcohol use, biomass exposure, pets and carpets at home, dust allergy or seasonal variation and no history of having tuberculosis or contact with tuberculosis patients. She doesn’t have asthma. She never smoked or had any other addictions. The patient had never been exposed to COVID and had been vaccinated against it.

Upon examination, she had a thin, lean appearance and an unhealthy face. Her initial blood pressure was 124/71 mmHg, heart rate was 92 beats per minute, and her respiratory rate was 22 breaths/min. She didn't have a temperature. General physical examination showed pallor and lymph nodes were not palpable. There were no clubbing, cyanosis, volume depletion and pedal edema. Chest auscultation revealed normal vesicular breathing on both sides and normal heart sound with no appreciable murmur. Abdomen was soft, not tender and there was no visceromegaly. The nervous system examination was unremarkable.

She had been through multiple physicians before and had completed different courses of antibiotics. She also had received two course of prednisolone (30mg/day for 5 and 7 days, respectively two weeks apart). Despite this, there was no significant improvement in her symptoms. Therefore, she was then admitted to our tertiary care hospital, where an extensive work-up was performed. Laboratory parameters of the patient is shown in Table 1.

**Fig. 1:**
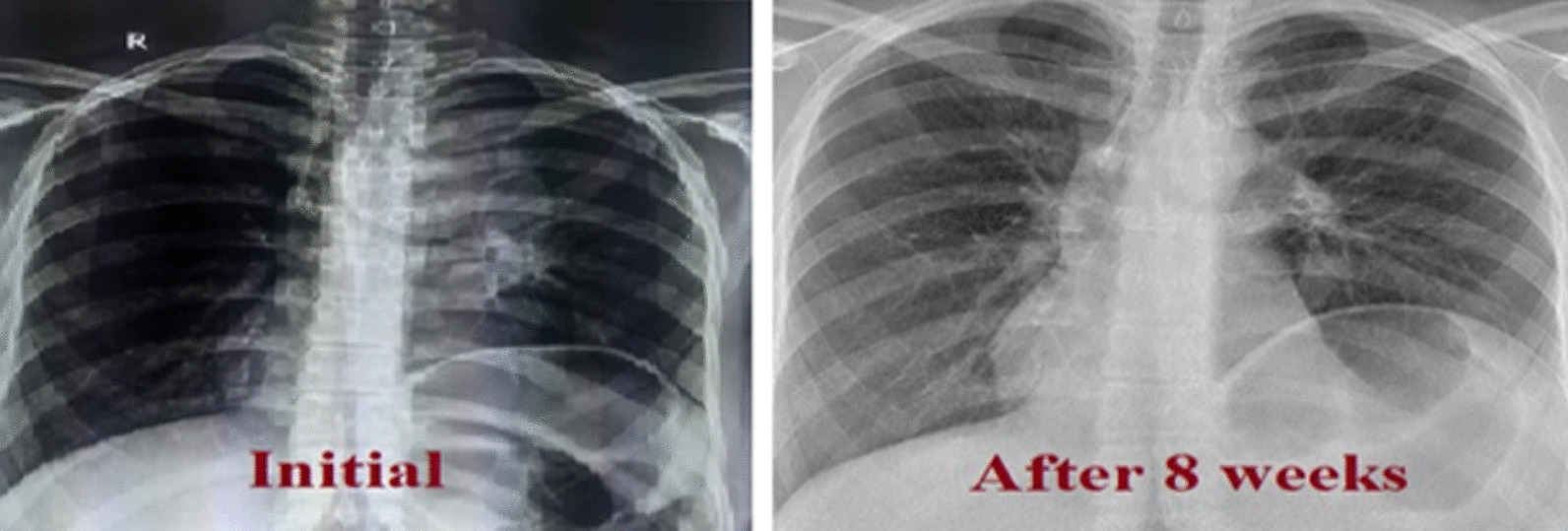
Chest X-rays showing prominent left bronchoalveolar marking and elevation of left hemidiaphragm

Chest x-ray showed prominent bronchoalveolar markings on her left side (Fig. 1)

**Table 1:** Laboratory parameters of the patient on first day of admission

	Patient’s value	Reference range
Hemoglobin (g/dL)	12.1	11–14.5
White Cell Count (× 10^9^/μL)	**14.4**	4–10
Platelets (× 10^9^/μL)	189	154–433
Serum Creatinine (mg/dL)	0.6	0.6–1.0
Sodium (mmol/L)	138	136–145
Potassium (mmol/L)	3.9	3.5–5.1
Chloride (mmol/L)	100	98–107
Bicarbonate (mmol/L)	24	20—31
SGPT (U/L)	35	< 35
SGOT (U/L)	30	< 35
C reactive protein (mg/dL)	**29**	0–14
Serum IgA (g/L)	2.1	0.8–3.0
Serum IgG (g/L)	9.4	6.0–16.0
Serum IgM (g/L)	1.1	0.4–2.5
Serum IgE (kU/L)	4.35	1.5–144
Beta d-glucan (pg/mL)	**183**	Positive > 80
Serum galactomannan	0.51	Positive: single sample > 0.7 or two consecutive sample > 0.5
HIV ELISA	Negative	
ANA	Negative	
AMA	Negative	
ASMA	Negative	
COVID-19 PCR	Negative	
HbA1c (%)	5.8%	

Bold are those which are abnormal lab parameters

*SGPT* serum glutamic pyruvic transaminase, *SGOT* serum glutamic-oxaloacetic transaminase, *HIV* human immunodeficiency virus, *ELISA* enzyme-Linked immunosorbent assay, *ANA* antinuclear antibodies, *AMA* anti-mitochondrial antibody, *ASMA* anti-smooth muscle antibody, *PCR* polymerase chain reaction

**Fig. 2:**
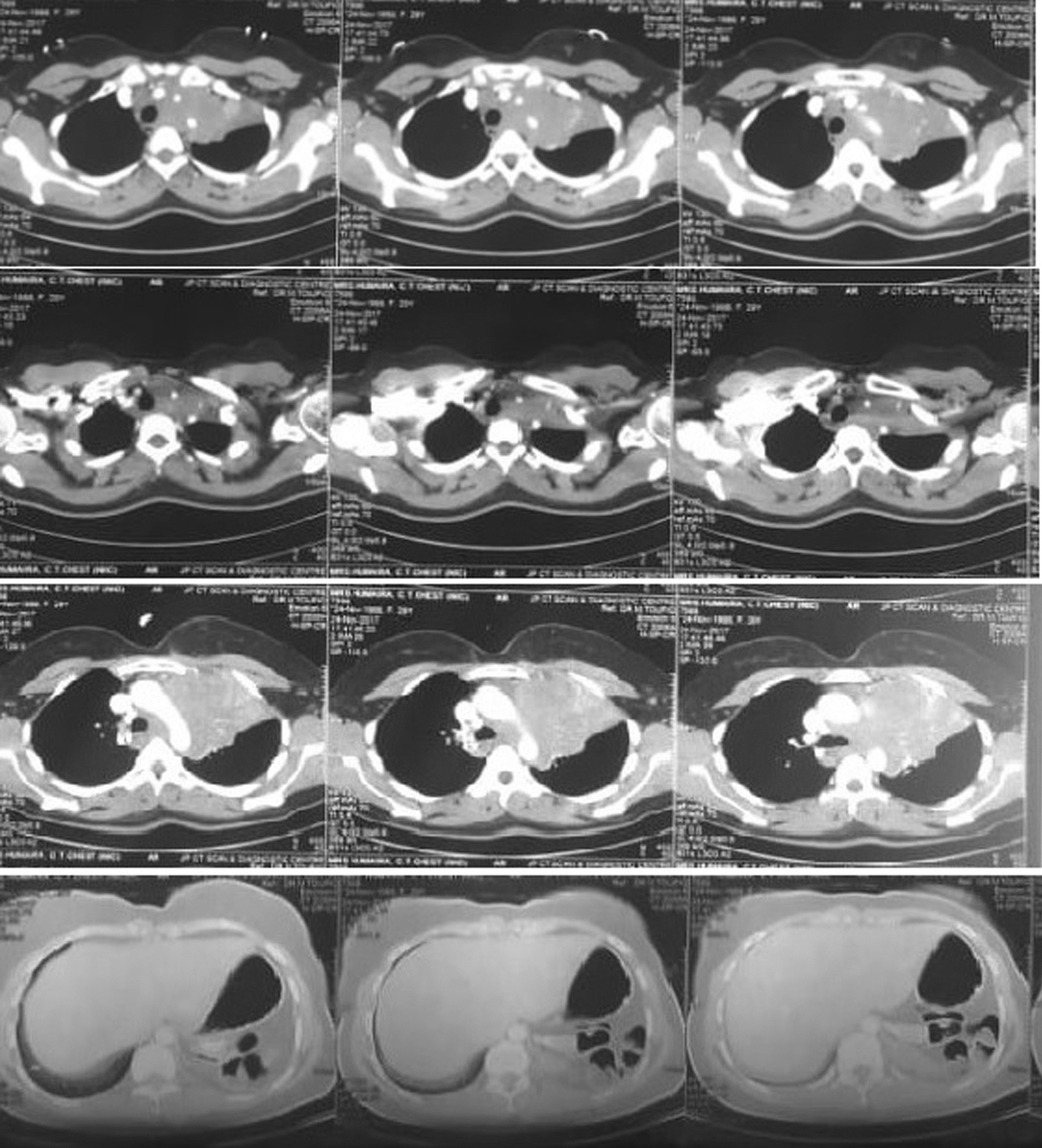
Cross sectional image of computed tomography scan of the chest showing an ill-defined hypodense lesion in the left upper lobe of lung

Computed tomography scan of the chest showed ill-defined hypodense lesion in left upper lobe of lung which is inseparable from superior mediastinum, lobulated outer contour with encasement of the left subclavian, common carotid and brachiocephalic artery, partial collapse and consolidation of left upper lobe of lung, mild to moderate pericardial effusion, multiple mediastinal lymph node enlargement. (Fig. 2) Thickening of the left adrenal gland by 2 cm. In addition, enlargement of the lymph nodes in the lesser sac of the body and in the antrum of stomach. These findings raise suspicion of tuberculosis versus lymphoma.

She underwent bronchoscopy and lavage, and empirical antibiotics were continued. The result of the lavage was negative for gram staining, culture, ZN staining for AFB smear, culture and gene xpert. Her fungal smear was positive for numerous septate hyphae. The histopathology report showed chronic granulomatous inflammation with septal fungal hyphae, suggesting aspergillosis. Later, her culture grew species of Aspergillus. She was started on voriconazole and all other medications were stopped. She showed a drastic response and her daily functional activities were regained. She started to gain weight and her appetite improved. Within two months of the treatment, her symptoms resolved, and her quality of life was significantly improved resulting in ease of performing her daily routine activities almost equal to normal.

## Discussion

We are reporting a case of invasive aspergillosis in a young lady who had multiple pulmonary nodules and mediastinal and abdominal involvement with invasive fungal infections.

The spectrum of aspergillosis is variable and can present in different forms. It includes non-invasive forms (i.e., allergic bronchopulmonary aspergillosis and chronic pulmonary aspergillosis) and invasive aspergillosis [2]. Invasive aspergillosis is usually identified in immunocompromised patients but can present in normal individuals. The disease usually progresses very rapidly and within a few weeks. The most common risk factor in immunocompromised individuals is neutropenia. However, prolonged corticosteroid use has been reported to promote aspergillosis in otherwise healthy individuals [7]. We also observed an immunocompetent female with invasive aspergillosis. However, she used corticosteroids for a shorter period of time. So the immunosuppressive effect of corticosteroids was not an attributable risk factor in her case.

Invasive pulmonary aspergillosis clinically mimics bronchopneumonia, so diagnosis is challenging [8]. Extra-pulmonary aspergillosis is gaining more importance and usually disseminated aspergillosis has been associated with GI aspergillosis [9]. Our patient also presented with lower respiratory tract symptoms and was later diagnosed as disseminated fungal infection. She not only had mediastinal and abdominal lymph node involvement but also had adrenal gland involvement.

Classical radiographic sign which raises suspicion of aspergillosis is a clear halo sign, but is not confirmatory and cannot be present in other cases [3]. Various elements are now used to diagnose invasive aspergillosis. These include identifying risk factors present in the patient, mycological laboratory to grow cultures, biomarkers like beta D glucan and galactomanan and histopathology/cytology confirming septate hyphae [10]. Our patient also required invasive work-up with bronchoscopy to confirm granulomatous inflammation with septate hyphae. And the lavage fungal smear was also positive and culture grew species of aspergillus.

Initiation of antifungal therapy early in the disease course had been associated with a better prognosis. Randomized control trial showed voriconazole as a drug of choice in aspergillosis. Voriconazole had better penetration and is also effective in invasive aspergillosis [11]. Other options include amphotericin B, caspofungin and itraconazole [8] We also used voriconazole in our patient and she showed a complete resolution of symptoms. Within two months of the treatment, her daily routine activities were almost equal to normal.

With this, we didn’t find any clear cut evidence of immunosuppression in a young healthy lady who acquired invasive aspergillosis. Probably, a short course of corticosteroid was a turning point and made her vulnerable to this type of infection.

## Conclusion

This case highlights the diagnosis of aspergillosis in an immunocompetent individual presenting with disseminated nodular lesions across the lungs, mediastinum, and abdomen. Clinicians should maintain a high index of suspicion for aspergillosis in cases of non-resolving pneumonia and disseminated nodular lesions, even in patients lacking traditional predisposing factors.

## Data Availability

All data generated or analysed during this study are included in this published article.

## References

[1] Patterson TF, Thompson GR 3rd, Denning DW, *et al*. Practice guidelines for the diagnosis and management of Aspergillosis: 2016 update by the Infectious Diseases Society of America. Clin Infect Dis. 2016;63(4):e1–60. 27365388 10.1093/cid/ciw326 PMC4967602

[2] Cadena J, Thompson GR 3rd, Patterson TF. Invasive Aspergillosis: current strategies for diagnosis and management. Infect Dis Clin North Am. 2016;30(1):125–42. 26897064 10.1016/j.idc.2015.10.015

[3] Moreno-Gonzalez G, Ricart de Mesones A, Tazi-Mezalek R, *et al*. Invasive pulmonary Aspergillosis with disseminated infection in immunocompetent patient. Can Respir J. 2016;2016:7984032. 27445566 10.1155/2016/7984032 PMC4904540

[4] Kartik M, Kanala A, Sunilnadikuda LNU, *et al*. Invasive mediastinal Aspergillosis in immunocompetent male with invasion of left atrium and hilar structures. Indian J Crit Care Med. 2017;21(6):408–11. 28701850 10.4103/ijccm.IJCCM_18_17 PMC5492746

[5] Kosmidis C, Denning DW. The clinical spectrum of pulmonary aspergillosis. Thorax. 2015;70(3):270–7. 25354514 10.1136/thoraxjnl-2014-206291

[6] Chen L, Han X, Li Y, *et al*. Invasive pulmonary aspergillosis in immunocompetent patients hospitalised with influenza A-related pneumonia: a multicenter retrospective study. BMC Pulm Med. 2020;20:239. 32907585 10.1186/s12890-020-01257-w PMC7479745

[7] Kosmidis C, Denning DW. Republished: the clinical spectrum of pulmonary aspergillosis. Postgrad Med J. 2015;91(1077):403–10. 26187954 10.1136/postgradmedj-2014-206291rep

[8] Mohammed AP, Dhunputh P, Chiluka R, *et al*. An unusual case of invasive aspergillosis in an immunocompetent individual. BMJ Case Rep. 2015;2015. 10.1136/bcr-2015-210381 PMC4488615 26123468

[9] Cha SA, Kim MH, Lim TS, *et al*. Invasive primary colonic Aspergillosis in the immunocompetent host without classical risk factors. Yonsei Med J. 2015;56(5):1453–6. 26256995 10.3349/ymj.2015.56.5.1453 PMC4541682

[10] Rabagliati R. Update in the diagnostic and therapeutic approach of invasive aspergillosis in adult population. Rev Chilena Infectol. 2018;35(5):531–44. 30725000 10.4067/s0716-10182018000500531

[11] Steinbach WJ, Juvvadi PR, Fortwendel JR, *et al*. Newer combination antifungal therapies for invasive aspergillosis. Med Mycol. 2011;49(Suppl 1):S77-81. 20608784 10.3109/13693786.2010.499374 PMC4442698

